# Pharmacokinetic Interaction between Sorafenib and Atorvastatin, and Sorafenib and Metformin in Rats

**DOI:** 10.3390/pharmaceutics12070600

**Published:** 2020-06-28

**Authors:** Agnieszka Karbownik, Danuta Szkutnik-Fiedler, Andrzej Czyrski, Natalia Kostewicz, Paulina Kaczmarska, Małgorzata Bekier, Joanna Stanisławiak-Rudowicz, Marta Karaźniewicz-Łada, Anna Wolc, Franciszek Główka, Edmund Grześkowiak, Edyta Szałek

**Affiliations:** 1 Department of Clinical Pharmacy and Biopharmacy, Poznań University of Medical Sciences, 61-861 Poznań, Poland; dszkutnik@ump.edu.pl (D.S.-F.); kostewicz.natalia@gmail.com (N.K.); p.kaczmarska95@gmail.com (P.K.); gosia.bekier01@gmail.com (M.B.); grzesko@ump.edu.pl (E.G.); eszalek@ump.edu.pl (E.S.); 2 Department of Physical Pharmacy and Pharmacokinetics, Poznań University of Medical Sciences, 60-781 Poznań, Poland; aczyrski@ump.edu.pl (A.C.); mkaraz@ump.edu.pl (M.K.-Ł.); glowka@ump.edu.pl (F.G.); 3 Department of Gynecological Oncology, University Hospital of Lord’s Transfiguration, 60-569 Poznań, Poland; stanisl@interia.pl; 4 Department of Animal Science, Iowa State University, Ames, IA 50011, USA; awolc@iastate.edu; 5 Hy-Line International, Research and Development, Dallas Center, IA 50063, USA

**Keywords:** sorafenib, sorafenib N-oxide, atorvastatin, metformin, pharmacokinetics, drug–drug interaction

## Abstract

The tyrosine kinase inhibitor sorafenib is the first-line treatment for patients with hepatocellular carcinoma (HCC), in which hyperlipidemia and type 2 diabetes mellitus (T2DM) may often coexist. Protein transporters like organic cation (OCT) and multidrug and toxin extrusion (MATE) are involved in the response to sorafenib, as well as in that to the anti-diabetic drug metformin or atorvastatin, used in hyperlipidemia. Changes in the activity of these transporters may lead to pharmacokinetic interactions, which are of clinical significance. The study aimed to assess the sorafenib−metformin and sorafenib−atorvastatin interactions in rats. The rats were divided into five groups (eight animals in each) that received sorafenib and atorvastatin (I_SOR+AT_), sorafenib and metformin (II_SOR+MET_), sorafenib (III_SOR_), atorvastatin (IV_AT_), and metformin (V_MET_). Atorvastatin significantly increased the maximum plasma concentration (C_max_) and the area under the plasma concentration–time curve (AUC) of sorafenib by 134.4% (*p* < 0.0001) and 66.6% (*p* < 0.0001), respectively. Sorafenib, in turn, caused a significant increase in the AUC of atorvastatin by 94.0% (*p* = 0.0038) and its metabolites 2−hydroxy atorvastatin (*p* = 0.0239) and 4−hydroxy atorvastatin (*p* = 0.0002) by 55.3% and 209.4%, respectively. Metformin significantly decreased the AUC of sorafenib (*p* = 0.0065). The AUC ratio (II_SOR+MET_ group/III_SOR_ group) for sorafenib was equal to 0.6. Sorafenib did not statistically significantly influence the exposure to metformin. The pharmacokinetic interactions observed in this study may be of clinical relevance in HCC patients with coexistent hyperlipidemia or T2DM.

## 1. Introduction

Hepatocellular carcinoma (HCC) is one of the most common primary malignant liver tumors whose morbidity is on the rise [1]. Both abnormal lipid metabolism and type 2 diabetes mellitus (T2DM) not only notably increase the risk of developing HCC but also accelerate the progress of existing disease and make treatment more challenging [2,3,4].

Sorafenib is the first-line treatment for patients with HCC. This tyrosine kinase inhibitor (TKI) is transported in the plasma predominantly in a protein-bound form (99.5%). It appears in the systemic circulation mostly unchanged (approximately 70−85%) or as a sorafenib N−oxide (SR_NO; approximately 9−16%). Sorafenib penetrates the liver possibly via organic cation transporter−1 (OCT1, gene *SLC22A1*) and organic anion transporting polypeptide 1B1/3 (OATP1B1/3; *SLCO1B1/3*) in humans, and in *Rattus norvegicus*, via Oatp1b2 (*Slco1b2*) as well. Then, it is metabolized by CYP3A4 to pharmacologically active SR_NO and inactive sorafenib glucuronide (SR_G). SR_G is secreted within the bile into the intestines through multidrug resistance-associated protein 2 (MRP2; *ABCC2*, *Abcc2*), where it can be excreted or converted into a parent compound and re-absorbed into the systemic circulation. Sorafenib is also carried by efflux transporters—P−glycoprotein (P−pg; *ABCB1*, *Abcb1a*), breast cancer resistance protein (BCRP; *ABCG2*, *Abcg2*) and MRP3 (*ABCC3*, *Abcc3*)—which also transport SR_G from the hepatocytes to the blood [5,6,7,8].

Atorvastatin normalizes the profile of lipids, due to the inhibition of HMG−CoA reductase, which is the rate-limiting step in cholesterol synthesis. It is metabolized by CYP3A4 and a substrate for protein transporters such as OATPs, BCRP, and P−gp [9]. An in vitro study performed on tumor cell lines revealed enhanced cytotoxic effects after the co-use of statins (e.g., atorvastatin) with sorafenib, which probably occurs due to the reduction of sorafenib elimination (shown by the clearance decrease to 0.48 L/h) [10]. A pre-clinical study showed that atorvastatin inhibits Rho-kinase, which is involved in the activation of hepatic stellate cells, which play a significant role in liver fibrosis progression. [11,12]. The addition of statins notably (*p* < 0.001) improved the mean progression-free survival (mPFS) and the mean overall survival (mOS of patients with lung cancer treated with EGFR-TKI (gefitinib or erlotinib) [13], which encourages further research on the impact of statins on oncology treatment.

Metformin is an oral anti-diabetic drug that lowers the blood glucose level mainly by reducing gluconeogenesis and glycogenolysis—most likely by AMP-activated protein kinase (AMPK) stimulation. Metformin is a non-metabolized strong base (a cation at physiological pH), which is taken up in the intestine into the bloodstream via plasma membrane monoamine transporter (PMAT; *SLC29A4*, *Slc29a4*) and then actively transported into hepatocytes and renal tubular epithelial cells by OCT1, OCT2 (*SLC22A2*, *Slc22a2*), and probably OCT3 (*SLC22A3*). However, its plasma protein-bound fraction is considered negligible. It is secreted in an unchanged form from these cells into the bile or urine, through multidrug and toxin extrusion protein transporter-1 (MATE1; *SLC47A1*, *Slc47a1*) and MATE2 (*SLC47A2*) [14,15,16]. In the last few years, a growing number of studies have investigated the anti-cancer properties of metformin and TKIs [17,18,19,20]. In vitro studies suggest that the addition of metformin may significantly decrease the dose of sorafenib needed to induce the equal inhibition of both the growth and colony and tumor formation of HT74 anaplastic thyroid carcinoma cells [21]. Previous clinical trials suggest that the sorafenib+metformin therapy of patients with advanced HCC may lead to a worse outcome, especially in comparison with, for example, sorafenib+insulin treatment—probably due to the promotion of the development of sorafenib resistance caused by the chronic use of metformin [22,23,24]. Those findings suggest that there might be also co-existing pharmacokinetic sorafenib−metformin interaction. Additionally, drug–drug interactions (DDIs) of clinical significance involve OCT and MATE transporters. Studies have revealed TKI (i.e., imatinib)−metformin DDIs that affected the efficacy and toxicity of metformin [15,25]. Since metformin’s pharmacokinetics and, hence, pharmacological effects are dependent on the activity of drug transporters (OCTs, MATEs, and PMAT), drugs that are inhibitors or inducers of these transporters may interfere with the distribution of metformin and ultimately affect both the plasma and intracellular concentrations of this drug. It is known that at high concentrations, especially in patients with widespread metastases, metformin can cause lactic acidosis, a life-threatening adverse reaction [16].

Due to the incidence of HCC in patients treated for hyperlipidemia and/or T2DM, and since the modulation of transporter protein activity may lead to pharmacokinetic interactions resulting in increased or decreased absorption of the drug, the aim of the study was to assess the influence of metformin and atorvastatin on the pharmacokinetics of sorafenib (and its active metabolite) and vice versa.

## 2. Materials and Methods

All applicable international, national, and/or institutional guidelines for the care and use of animals were followed. Animals were given a standard diet and water ad libitum, and the experimental protocol for this study was approved by the Local Ethics Committee (Number 02/2019, from 1 March 2019), Poznań University of Life Sciences, Department of Animal Physiology and Biochemistry, Wołyńska 35 Str., 60-637 Poznań, Poland.

### 2.1. Animal Experiments

Adult, healthy, fed male Wistar rats (weight, 475−530 g) were divided into five groups—two study groups and three control groups. The study groups—I_SOR+AT_ and II_SOR+MET_—received sorafenib, along with atorvastatin or metformin, respectively. The control groups—III_SOR_, IV_AT_, and V_MET_—received sorafenib, atorvastatin, or metformin, respectively, along with 1 mL of vehicle. The groups of rats did not differ significantly in terms of body mass. Sorafenib (100 mg/kg body weight (b.w.) [26]; Nexavar^®^, batch number BXHT61, Bayer AG, Leverkusen, Germany), atorvastatin (20 mg/kg b.w. [27]; Tulip^®^, batch number JK9440, Sequoia Capital, Menlo Park, California, USA), and metformin (100 mg/kg b.w. [28]; Metformax^®^, batch number 16518316, Teva Pharmaceutical Industries Ltd., Petach Tikwa, Izrael) were administrated directly into the stomachs of live animals using a gastric probe (1 mL of each solution). A 10% dimethyl sulfoxide (DMSO) stock for sorafenib tosylate and 0.9% sodium chloride for atoravastatin and metformin were used to prepare the drug solutions. Blood samples were collected before administration and after dosing at the following time points: 0.5, 1, 2, 3, 4, 5, 6, 7, 8, 10, 12, 24, 30, 48, 72, and 96 h (for sorafenib); 0.083, 0.25, 0.5, 0.75, 1, 2, 4, 6, and 12 h (for atorvastatin); and 0.083, 0.25, 0.5, 1, 1,5, 2, 4, 6, 8, 10, and 24 h (for metformin). The plasma was separated by centrifugation at 2880× *g* for 10 min at 4 °C and stored at −80 °C until drug analysis. One subject, assigned to the II_SOR+MET_ group, was excluded from analysis due to an insufficient blood sample volume.

### 2.2. Reagents

All the reagents used for HPLC-UV and the UPLC-MS/MS assays are listed in Table 1.

### 2.3. HPLC-UV Assays

The concentrations of sorafenib, SR_NO, and metformin were assayed using high-performance liquid chromatography (HPLC) methods with ultraviolet (UV) detection (HPLC Waters 2695 Separations Module with autosampler, Waters 2487 Dual λ Absorbance Detector) [29,30].

Separation was achieved by the gradient elution of the mobile phase, from ammonium acetate (0.1 M, pH = 3.4 (adjusted with glacial acetic acid)) as eluent A to an acetonitrile eluent B, at a flow rate of 1.0 mL/min through a reversed-phase C8 column (Symmetry^®^ C8, 250 × 4.6 mm, 5.0 μm particle size) (Waters Corporation^®^, Milford, Massachusetts, USA). The linear gradient ran from 60% eluent A and 40% eluent B to 29% eluent A and 71% eluent B. The column temperature was maintained at 25 °C, the UV detection wavelength was set at 265 nm, and the injection volume was 20 μL; lapatinib was used as the internal standard (IS).

The metformin concentrations in rats’ plasma were determined according to the method described by Gabr et al. [30]. The conditions were as follows: the UV detection wavelength was 236 nm; a Symmetry^®^ C8, 250 × 4.6 mm, 5.0 μm-particle-size column from Waters was used; the column temperature was maintained at 25 °C; the mobile phase consisted of ammonium formate/acetonitrile (95:5, v/v); the pH of the mobile phase was 6.0; the flow rate was 1.0 mL/min; the volume of each injection was 20 µL; and the retention times for metformin and the internal standard (acetaminophen) were 3.24 and 9.27 min, respectively.

The calibration curve for sorafenib, SR_NO, and metformin was linear within the range of 0.025−5.0 µg/mL (r = 0.999), 0.025−0.50 µg/mL (r = 0.997), and 0.1−2.0 µg/mL (r = 0.996), respectively. The lower limit of quantification (LLOQ) was 0.025 µg/mL for sorafenib and SR_NO, and 0.1 µg/mL for metformin. The intra- and inter-day precision (coefficients of variation (CV) and accuracy (%bias) were as follows: CV < 12% and %bias ≤ 7.5% for sorafenib and SR_NO, and CV < 15% and %bias ≤ 15% for metformin.

### 2.4. UPLC-MS/MS Assay

The concentrations of atorvastatin, 2-hydroxy atorvastatin (2-OH AT), and 4-hydroxy atorvastatin (4-OH AT) were assayed using a UPLC-MS/MS method. The concentration of each of the working solutions was 10 μg/mL. The sample preparation involved liquid–liquid extraction. The dry residue was dissolved in 50 μL of the mobile phase, and 20 μL was injected into the UPLC Nexera system coupled to an LCMS−8030 Triple Quadrupole tandem mass spectrometer (Shimadzu Corp., Kioto, Japan). The analytes were separated in a Zorbax Plus C18 column (100 × 2.1 mm; 3.5 µm) (Agilent Technologies, Santa Clara, California, USA) at a column temperature of 40 °C. The mobile phase was a mixture of de-ionized water (A) and acetonitrile (B), both containing 0.1% (v/v) formic acid. The gradient was as follows: 0–2 min, linear from 50 to 70% B; 2–4 min, 70% B; 4–6 min, a return from 70 to 50% B; and a post-time of 4 min with 50% B for column equilibration. The mobile phase flow was set to 0.3 mL/min. The eluent from the HPLC column was introduced directly to the MS interface, using electrospray ionization in the positive ion mode. The MS parameters were as follows: interface temperature, 350 °C; DL temperature, 250 °C; heat block temperature, 400 °C; nebulizing gas flow, 2 L/min; and drying gas flow, 15 L/min. The electrospray needle voltage was 3.5 kV. The specific transitions for the analytes were monitored using the multiple reaction monitoring (MRM) mode. The most sensitive mass transition was monitored from m/z 558.8 to 440.1 (for atorvastatin), 547.9 to 440.2 (for 2-OH AT and 4-OH AT), and 481.7 to 258.0 (for rosuvastatin).

The UPLC−MS/MS method for atorvastatin, 2-OH AT, and 4-OH AT showed linearity in the concentration range of 0.4–200 ng/mL in plasma. The intra- and inter-assay precision values, expressed as relative standard deviations, were <18.7% for atorvastatin and 4-OH AT, and <14.0% for 2-OH AT. The intra- and inter-day accuracy of the method, expressed as the relative error, was <14.0, <6.1, and <10.2% for atorvastatin, 4-OH AT, and 2-OH AT, respectively.

### 2.5. Pharmacokinetic Evaluation

The Phoenix^®^ WinNonlin version 8.0 software (Certara, Princeton, New Jersey, USA) was used for the calculation of the following pharmacokinetic parameters:

- The elimination rate constant (k_e_);
- The absorption rate constant (k_a_);
- The elimination half-life (t_1/2_);
- The maximum plasma concentration (C_max_);
- The time to reach the C_max_ (t_max_);
- The total area under the concentration–time curve (AUC_0-t_ and AUC_0--∞_);
- The apparent plasma drug clearance (Cl/F);
- The apparent volume of distribution (V_d_/F).

The above-mentioned parameters underwent statistical analysis.

### 2.6. Statistical Analysis

The statistical analyses were performed using the Statistica software (Statistica, Tulsa, OK, USA) version 13.3 (for atorvastatin, 2-OH AT, 4-OH AT, and metformin) and SAS software (SAS Institute Inc., Cary, NC 27513, USA) version 9.4 (for sorafenib and SR_NO). Normality was estimated with the Shapiro–Wilk test. For sorafenib and SR_NO, analysis of variance (PROC GLM) was used to test the significance of the differences between the groups for variables without significant deviations from normality. Welch’s test was used when the assumption of homogeneity of variance was not met, and ANOVA was followed by post-hoc Tukey tests. For metformin, atorvastatin, and its metabolites, the differences between the normally distributed variables were determined with the Student’s t-test. For all the remaining variables, the Mann–Whitney U test was applied, and a *p*-value < 0.05 was considered significant.

## 3. Results

All the data are expressed as the mean values ± standard deviations (SD).

### 3.1. The Influence of Atorvastatin on the Pharmacokinetics of Sorafenib and SR_NO

**Figure 1:**
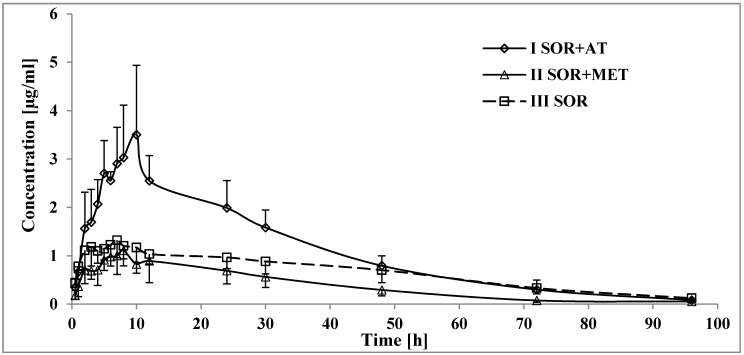
The sorafenib plasma concentration–time profiles in rats receiving sorafenib (III_SOR_), sorafenib+metformin (II_SOR+MET_), and sorafenib+atorvastatin (I_SOR+AT_).

After the administration of sorafenib with atorvastatin (I_SOR+AT_ group), the C_max_, AUC_0-t_, and AUC_0-∞_ of sorafenib increased by 134.6, 66.6, and 58.7%, respectively, compared with those in the III_SOR_ group (Table 2, Figure 1). The C_max_ of sorafenib occurred later (t_max_ increased by 116.9% and k_a_ decreased by 73.0%) in the I_SOR+AT_ group. The value of the t_1/2_ of sorafenib decreased in the presence of atorvastatin (the t_1/2_ was lower by 0.3-fold). However, there were no statistically significant differences for the k_el_ and Cl/F of sorafenib. V_d_/F was also comparable between the III_SOR_ and I_SOR+AT_ groups.

**Figure 2:**
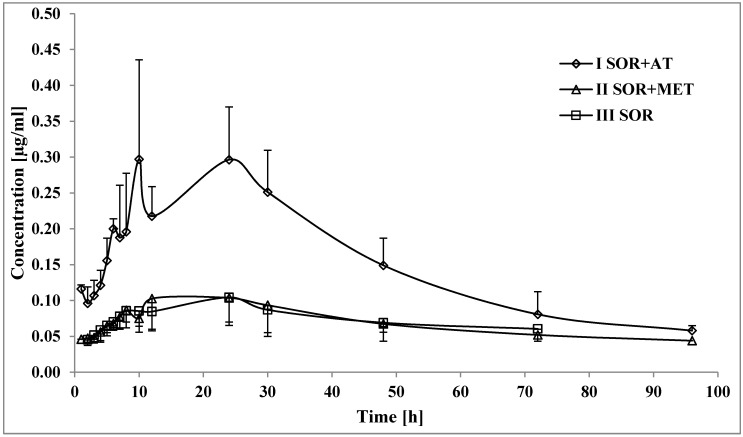
The sorafenib N-oxide plasma concentration–time profiles in rats receiving sorafenib (III_SOR_), sorafenib+metformin (II_SOR+MET_), and sorafenib+atorvastatin (I_SOR+AT_).

Atorvastatin increased the SR_NO C_max_, AUC_0-t_, and AUC_0-∞_ by 245.5, 242.4, and 89.5%, respectively (Table 2, Figure 2); the t_0.5_ of SR_NO in the I_SOR+AT_ group decreased by 0.4-fold. However, no statistically significant differences were revealed for k_el_ and t_max_.

### 3.2. The Influence of Sorafenib on the Pharmacokinetics of Atorvastatin, 2-OH AT, and 4-OH AT

**Figure 3:**
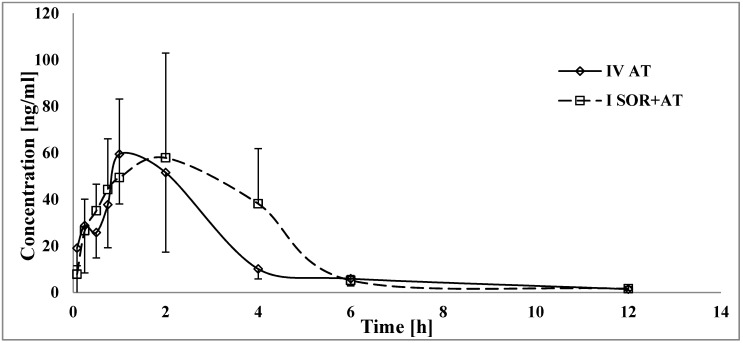
The atorvastatin plasma concentration–time profiles in rats receiving sorafenib (IV_AT_) and sorafenib+atorvastatin (I_SOR+AT_).

When atorvastatin and sorafenib were co-administered, the AUC_0-t_ and AUC_0--∞_ of atorvastatin increased by 94.0 and 93.2%, respectively (Table 3, Figure 3). In the group of rats receiving both drugs, atorvastatin t_max_ was longer than that in the IV_AT_ group. No statistically significant differences were revealed for C_max_, k_a_, k_el_, and t_1/2_.

**Figure 4:**
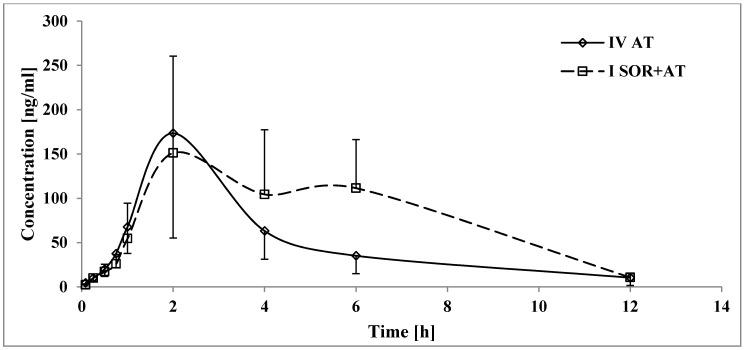
The 2-OH AT plasma concentration–time profiles in rats receiving sorafenib (IV_AT_) and sorafenib+atorvastatin (I_SOR+AT_).

**Figure 5:**
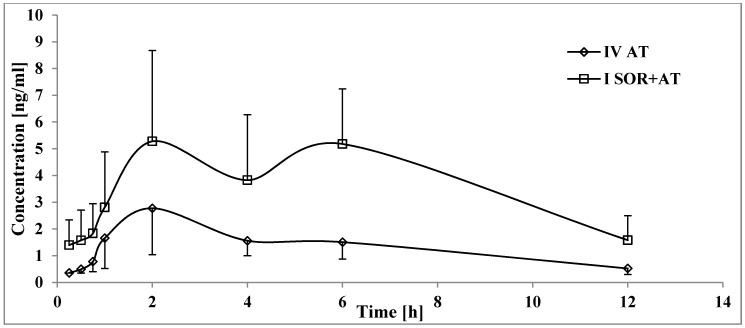
The 4-OH AT plasma concentration–time profiles in rats receiving sorafenib (IV_AT_) and sorafenib+atorvastatin (I_SOR+AT_).

The C_max_ of 2-OH AT was similar in both the atorvastatin and atorvastatin+sorafenib groups (Table 3, Figure 4). The exposure to 2-OH AT was significantly higher in the presence of sorafenib, which was reflected by increased values of AUC_0-t_ and AUC_0-∞_, but for AUC_0-∞_, there was no statistical significance. The ratios of AUC_0–t_ and AUC_0–∞_ (I_SOR+AT_ group/IV_AT_ group) for atorvastatin were increased by 0.5- and 0.4-fold, respectively. In the I_SOR+AT_ group, the t_max_ of 2-OH AT was longer than that in the IV_AT_ group, but there was no statistical significance. No statistically significant differences were revealed for k_el_ and t_1/2_. Sorafenib elevated the 4-OH AT C_max_ by 127.0% (Table 3, Figure 5). When atorvastatin and sorafenib were co-administered, the AUC_0-t_ and AUC_0-∞_ of 4-OH AT increased by 320.9 and 220.5%, respectively. There were no significant differences among the groups for the following pharmacokinetic parameters of 4-OH AT: k_el_, t_1/2_, and t_max_.

### 3.3. The Influence of Metformin on the Pharmacokinetics of Sorafenib and SR_NO

In the presence of metformin (II_SOR+MET_ group,) the sorafenib plasma AUC_0-t_ and AUC_0-∞_, were 44.0 and 45.1% lower than those in the III_SOR_ group (Table 2, Figure 1). Metformin decreased the k_a_ by 0.5-fold. However, the C_max_ and t_max_ of sorafenib were comparable. The clearance of sorafenib in the II_SOR+MET_ group was higher by 76.3% than that in the III_SOR_ group, but there were no significant differences for k_el_ and t_0.5_. Likewise, the V_d_/F of sorafenib did not differ significantly between the groups.

All the pharmacokinetic parameters of SR_NO in the II_SOR+MET_ and III_SOR_ groups were comparable (Table 2, Figure 2).

### 3.4. The Influence of Sorafenib on the Pharmacokinetics of Metformin

**Figure 6:**
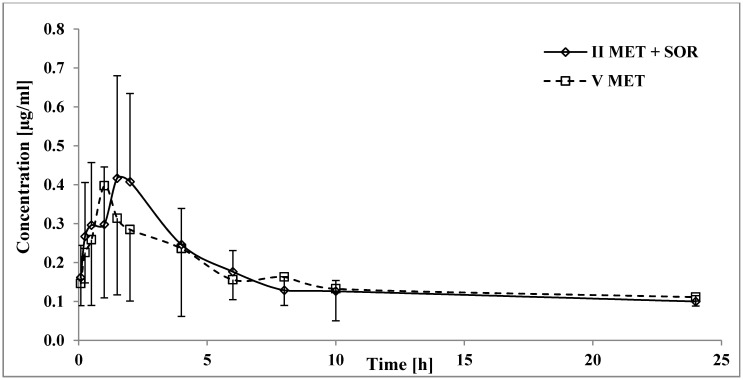
The metformin plasma concentration-time profiles in rats receiving metformin (V_MET_) and sorafenib+metformin (II_SOR+MET_).

Comparing both groups of animals (V_MET_ vs. II_SOR+MET_), statistically significant differences were observed only for t_max_, which was 71.39% higher in the _IISOR+MET_ group (Table 4, Figure 6). The values of the other pharmacokinetic parameters were comparable. High values of CV for k_a_ and k_el_ in the V_MET_ and II_SOR+MET_ groups, respectively, were observed. It may indicate significant inter-individual variability in animals in terms of metformin absorption and elimination.

## 4. Discussion

To properly evaluated our findings, it is important to note the limitations of the conducted studies. First of all, no animal model used in the experiment experienced HCC or T2DM, and as we know from our previous in vivo study with streptozotocin-induced diabetic Wistar rats receiving only sorafenib, T2DM has its impact upon exposure to sorafenib [31]. Additionally, the pharmacokinetic profile of sorafenib, metformin, and atorvastatin may differ between Wistar rats and humans, and the concentrations of sorafenib glucuronide were not measured. 

On the other hand, since we used non-diabetic rats, this condition did not enhance the exposure to sorafenib and SR_NO [31], and therefore, we were able to examine potential pharmacokinetic DDIs in this animal model. 

The doses of metformin (100 mg/kg) and atorvastatin (20 mg/kg) used in our study were selected based on available literature [27,28,32] and did not cause any adverse effects in healthy rats. A dose of atorvastatin of 20 mg/kg may cause liver injury in diabetic rats [27] but is safe for healthy rats [32].

Drug–drug interactions are clinically relevant in humans when changes in the AUC, C_max_, or clearance are greater than 25%, or there is a statistically significant influence on pharmacodynamics endpoints. For example, for metformin, glycosylated hemoglobin A1c (HbA1c) levels or oral glucose tolerance tests (OGTTs) may be relevant [16,33].

### 4.1. The Influence of Atorvastatin on the Pharmacokinetics of Sorafenib and SR_NO

We found that the co-administration of sorafenib and atorvastatin could lead to a higher risk of adverse reactions to sorafenib, such as hand-foot syndrome, alopecia, gastrointestinal disorders, a hypertensive crisis, or cardiotoxicity [34]. The almost doubling of exposure to sorafenib (*p* < 0.0001 for AUC_0-t_, and *p* = 0.0002 for AUC_0-∞_), along with the enhanced C_max_ (+134.6%, *p* < 0.0001) and k_el_ (+42.9%, *p* = 0.0637), most closely corresponds to Kalliokoski A. et al.’s results [35]. They concluded, based on a randomized crossover study with healthy volunteers (*n* = 24), that increased mean values of C_max_ and AUC for repaglinide (among those who received repaglinide and atorvastatin, 0.25 + 40 mg) were most likely the effect of OATP1B1-mediated hepatic uptake inhibition by atorvastatin [34]. Since sorafenib is highly protein-bound [33], there is the potential for it to be released from protein binding by atorvastatin, which would improve the unbound fraction concentration of the chemotherapeutic agent as well. Additionally, an in vitro study on a murine monocytic leukemia cell line overexpressing P-gp showed that the co-use of atorvastatin may result in the increased absorption of P-gp substrates (digoxin and verapamil) [36].

Additional sorafenib N-oxide exposure (Table 2) is probably a consequence of increased exposure to sorafenib. Considering the fact that both sorafenib and SR_NO exhibit inhibitory effects on CYP3A4 [37], and since atorvastatin itself is its substrate, the induction of CYP3A4 is highly unlikely. Since SR_NO exhibits pharmacological activity, the risk of toxicity is further enhanced.

### 4.2. The Influence of Sorafenib on the Pharmacokinetics of Atorvastatin, 2-OH AT, and 4-OH AT

Sorafenib+atorvastatin treatment may also lead to excessive side effects from atorvastatin, such as hyperglycemia, hepatitis, or musculoskeletal and connective tissue disorders [37], due to an almost doubling of the exposure to atorvastatin (Table 4). Since bioavailability (F) depends on the AUC_0-∞_ for extravascular administration and is directly proportional, a significant (*p* < 0.005) decrease in CL/F and V_d_/F is consistent with the reported data. Even though t_max_ was prolonged by 2.2-fold (*p* = 0.0419), there were no significant differences for the absorption (C_max_, k_a_) or elimination (k_el_ or t_1/2_) of atorvastatin. The lack of reduction of exposure to 2-OH AT and 4-OH AT among the I_SOR+AT_ group (Figure 4 and Figure 5) suggests that the CYP3A4-mediated metabolism of atorvastatin was not suppressed by sorafenib. In the light of those findings, interaction occurs most likely during atorvastatin distribution at the protein transporter level. The overlap between sorafenib and atorvastatin transporter-related activity suggests that the induction of efflux transporters (P-glycoprotein) and/or inhibition of atorvastatin hepatic uptake transporters (Oatp1b1/3) by sorafenib could play a significant role in this DDI [5,38].

Furthermore, as a consequence of the highly enhanced exposure to atorvastatin, the concentration of its metabolites (2-OH AT and 4-OH AT) should increase as well. However, decreases in the 2-OH AT/atorvastatin ratios for the AUC_0-t_ (−19.4%) and AUC_0-∞_ (−26.0%, *p* = 0.0312) (Table 3) may suggest a smaller increase upon exposure to 2-OH AT than expected. However, the differences in the AUC_0−∞_ for 2-OH AT and the AUC_0-t_ for 2-OH AT/atorvastatin ratio were statistically insignificant, and the C_max_ for 2-OH AT remains comparable. At the same time, the I_SOR+AT_ group was heavily exposed to 4-OH AT (*p* = 0.0002 for AUC_0-t_, *p* = 0.0001 for AUC_0-∞_, and *p* = 0.0019 for C_max_), which resulted in 2.5-, 1.6-, and 1.7-fold increases in the C_max_ (*p* = 0.0084), AUC_0-t_ (*p* = 0.0239), and AUC_0-∞_ (*p* = 0.0239) for the 4-OH AT/atorvastatin ratio, respectively, as well. The reported data suggest that the presence of sorafenib altered the proportions of 2-OH AT and 4-OH AT, in favor of 4-OH AT.

### 4.3. The Influence of Metformin on the Pharmacokinetics of Sorafenib and SR_NO

The co-administration of metformin with sorafenib significantly reduced and nearly halved rats’ exposure to the chemotherapeutic agent (Figure 1), and the increased CL/F value (*p* < 0.005) is consistent with the reported data (Table 2). Furthermore, when a 0.5-fold decrease in the absorption rate constant is compared to the inconsistent changes in elimination-related parameters, interaction at the absorption level seems to occur. Since the C_max_ and t_max_ values were comparable among the study and control groups, the possibility of interference within the protein-bound sorafenib fraction may be ruled out. Additionally, accelerated intestinal passage is one of the most common adverse reactions observed in patients during metformin treatment [39], and a clinically important interaction between a high-fat diet and sorafenib (resulting in a 30% decrease in sorafenib absorption) is known as well [34]. Although the defecation rate noted during the experiment was not unusual, it might interrupt the intestinal absorption and enterohepatic cycle, and eventually decrease sorafenib exposure.

Furthermore, despite the decreased exposure to sorafenib, all the pharmacokinetic parameters of SR_NO were still comparable in both the study and control groups (Table 2), which resulted in a 2.3-fold increased value of the AUC_0-t_ for SR_NO/sorafenib ratios in the II_SOR+MET_ group vs. the III_SOR_ group (*p* = 0.0002). Since SR_G plays a crucial role in the enterohepatic circulation of sorafenib [5], it is more likely that the increased SR_NO/sorafenib exposure ratios are a result of rapid intestinal transit, which shortens the time for SR_G transformation to sorafenib and sorafenib re-absorption, than a result of CYP3A4-mediated DDIs. However, sorafenib itself has shown CYP3A4-inhibitory properties in an in vitro study [37,40], and therefore, metformin could promote the CTP3A4-mediated pathway by alleviating the obstacles (by sorafenib concentration reduction). However, this metabolization hypothesis seems to be less established, especially considering that the direct impact of metformin on CYP3A4 activity remains largely unknown [41]. Although the pharmacological activity of sorafenib N-oxide and sorafenib are comparable [34], a halved exposure to sorafenib carries the risk of reduced, unsatisfactory responses to oncological treatment.

### 4.4. The Influence of Sorafenib on the Pharmacokinetics of Metformin

It has been shown that there are several drugs, inhibitors, that can cause an increase in the C_max_ and AUC of metformin, e.g., cimetidine (inhibits MATE1 in the kidneys) [42], cephalexin [43], topiramate [44], trimethoprim [45] (reduces the clearance of metformin), vandetanib (inhibits OCT2) [46], and pyrimethamine (competitively inhibits both MATE1 and MATE2) [47].

However, in our study, sorafenib did not significantly affect the total exposure to metformin in rats (Figure 6), and the C_max_ values of metformin in the V_MET_ and the II_SOR+MET_ groups were comparable (Table 4). After the combined use of these two drugs, only an increase in the t_max_ of metformin (by 71.39%, *p* = 0.0069) was observed. Those data suggest that DDIs during the absorption process may occur. Metformin is known for its saturable absorption and prolongs t_max_ due to its co-administration with food [14,39]. Since both PMAT and OCT1 transporters participate in the intestinal uptake of metformin, and sorafenib is still considered as an OCT1 substrate, these proteins might be a direct cause of metformin−sorafenib DDI [5,14].

Studies have reported that metformin partitions into erythrocytes, which are most likely its second compartment [14]. However, since the C_max_ values remain comparable between the study and control groups, interference within this fraction is insignificant.

## 5. Conclusions

Statins are commonly used by elderly patients that usually also struggle with other conditions, including kidney and liver disorders (these organs are important for proper xenobiotic elimination). Since that population is at a higher risk of presenting with cancer, further pharmacokinetic studies of sorafenib−atorvastatin interaction in patients are needed. Moreover, plasma sorafenib and SR_NO monitoring during sorafenib−atorvastatin therapy might be beneficial for preventing sorafenib overdose. Since 2-OH AT and 4-OH AT are together responsible for approximately 70% of the therapeutic activity of atorvastatin [32], and its profiles were strongly affected by the presence of sorafenib, it is also important to evaluate the influence of sorafenib−atorvastatin therapy on its pharmacokinetics in future clinical studies as well.

It can be assumed that in clinical practice, the combined use of sorafenib and metformin in HCC therapy will probably not result in an increase in the plasma levels of metformin and, thus, the risk of its side effects. It should also be noted that genetic variation in OCTs, MATEs, and PMAT transporter genes may bias the metformin plasma and intracellular levels and, hence, the patient’s response to metformin [16]. At the same time, there is a growing need to clarify the potential impact of metformin on sorafenib’s and sorafenib N-oxide’s pharmacokinetics, both in patients and possibly with a suitable time interval between the administration of those two drugs. However, it should be remembered that altered drug pharmacokinetics are not always translatable into significant pharmacodynamic changes. Further clinical studies should investigate pharmacokinetic relationships between drugs, in parallel with the pharmacological effect of combined therapies, as they may occur in patients as well.

## Figures and Tables

**Table 1:** List of all reagents for HPLC-UV and UPLC-MS/MS assays.

Reagent	CAS Number
1-butanol ^1^	71-36-3
2-hydroxy atorvastatin dihydrate monosodium salt ^2^	214217-86-4
4-hydroxy atorvastatin disodium salt ^2^	1276537-18-8
acetaminophen ^3^	103-90-2
acetonitrile ^3^	75-05-8
ammonium acetate ^3^	631-61-8
ammonium formate ^3^	540-69-2
atorvastatin calcium salt trihydrate ^2^	344423-98-9
dimethyl sulfoxide ^3^	67-68-5
ethyl acetate ^3^	141-78-6
formic acid ^3^	64-18-6
lapatinib ^3^	231277-92-9
metformin hydrochloride ^3^	1115-70-4
methanol ^3^	67-56-1
n-heptane ^4^	142-82-5
sodium hydroxide ^3^	67-56-1
sodium hydroxide microprills ^5^	1310-73-2
sorafenib ^6^	284461-73-0
sorafenib N-oxide ^6^	583840-03
rosuvastatin calcium ^3^	147098-20-2
ultrapure water (deionized, distilled, and filtered through Direct Q3 system) ^4^	7732-18-5

^1^ purchased from CHEMPUR (Piekary Śląskie, Poland); ^2^ purchased from Santa Cruz Biotechnology, Inc. (Dallas, TX, USA); ^3^ purchased from Sigma-Aldrich (Saint Louis, MO, USA); ^4^ purchased from Merck Millipore (Burlington, MA, USA); ^5^ purchased from POCH S.A. (Gliwice, Poland); ^6^ purchased from LGC (Teddington TW11 0LY, UK).

**Table 2:** Plasma pharmacokinetic parameters of sorafenib and its metabolite N-oxide after the oral administration of a single dose of sorafenib (100 mg/kg b.w.) to the III_SOR_ group, sorafenib+atorvastatin (100 mg/kg b.w. + 20 mg/kg b.w.) to the I_SOR+AT_ group, and sorafenib+metformin (100 mg/kg b.w. + 100 mg/kg b.w.) to the II_SOR+MET_ group.

Pharmacokinetic Parameters ^1^	III_SOR_ (*n* = 8)	I_SOR+AT_ (*n* = 8)	II_SOR+MET_ (*n* = 7)	*p*-Value I_SOR+AT_ vs. III_SOR_	*p*-Value II_SOR+MET_ vs. III_SOR_
**Sorafenib**					
C_max_ (µg/mL)	1.56 ± 0.35 (22.6)	3.66 ± 1.21 (33.1)	1.27 ± 0.38 (30.2)	<0.0001	0.7499
AUC_0-t_ (µg × h/mL)	62.83 ± 16.14 (25.7)	104.67 ± 18.90 (18.1)	35.16 ± 8.36 (23.8)	<0.0001	0.0065
AUC_0--∞_ (µg × h/mL)	67.05 ± 16.70 (24.9)	106.41 ± 19.33 (18.2)	36.79 ± 8.96 (24.4)	0.0002	0.0041
t_max_ (h)	5.13 ± 2.17 (42.3)	11.13 ± 5.54 (49.8)	7.43 ± 2.29 (30.9)	0.0117	0.4722
k_a_ (h^−1^)	0.74 ± 0.31 (42.5)	0.20 ± 0.11 (53.2)	0.35 ± 0.23 (66.8)	0.0005	0.0106
k_el_ (h^−1^)	0.035 ± 0.01 (30.3)	0.05 ± 0.02 (30.6)	0.05 ± 0.01 (24.4)	0.0637	0.3306
t_1/2_ (h)	21.89 ± 7.79 (35.6)	14.70 ± 3.17 (21.5)	16.33 ± 3.72 (22.8)	0.0372	0.1386
Cl/F (L/h × kg)	0.80 ± 0.22 (27.1)	0.48 ± 0.09 (18.3)	1.41 ± 0.50 (35.3)	0.1118	0.0026
V_d_/F (L)	25.30 ± 11.59 (45.8)	9.98 ± 2.48 (24.9)	34.15 ± 18.74 (54.9)	0.0570	0.3716
**Sorafenib N-oxide**					
C_max_ (µg/mL)	0.11 ± 0.02 (21.8)	0.38 ± 0.09 (24.0)	0.13 ± 0.04 (31.5)	<0.0001	0.8981
AUC_0-t_ (µg × h/mL)	4.10 ± 1.56 (38.1)	14.04 ± 2.11 (15.1)	5.49 ± 2.39 (43.6)	<0.0001	0.4029
AUC_0-∞_ (µg × h/mL)	8.61 ± 2.19 (25.4)	16.32 ± 2.50 (15.3)	8.55 ± 2.36 (27.5)	<0.0001	0.9988
t_max_ (h)	16.38 ± 8.21 (50.1)	19.50 ± 8.12 (41.7)	21.43 ± 6.80 (31.7)	0.7055	0.4363
k_el_ (h^−1^)	0.016 ± 0.010 (60.9)	0.028 ± 0.009 (31.4)	0.018 ± 0.009 (51.9)	0.0638	0.9473
t_1/2_ (h)	53.31 ± 25.23 (47.3)	27.04 ± 8.49 (31.4)	45.98 ± 20.71 (45.0)	0.0347	0.7487
**Sorafenib N-oxide/Sorafenib**					
C_max_ (µg/mL)	0.07 ± 0.02 (26.8)	0.11 ± 0.03 (26.9)	0.10 ± 0.03 (26.0)	0.0410	0.1231
AUC_0-t_ (µg × h/mL)	0.07 ± 0.02 (37.1)	0.14 ± 0.02 (12.7)	0.16 ± 0.06 (35.6)	0.0026	0.0002
AUC_0--∞_ (µg × h/mL)	0.14 ± 0.05 (38.1)	0.16 ± 0.03 (16.7)	0.25 ± 0.11 (42.2)	0.8226	0.0108

C_max_, maximum observed plasma concentration; AUC_0-t_, area under the plasma concentration–time curve from zero to the time of last measurable concentration; AUC_0--∞_, area under the plasma concentration–time curve from zero to infinity; t_max_, time to the first occurrence of C_max_; k_a_, absorption rate constant; k_el_, elimination rate constant; t_1/2_, half-life in the elimination phase; Cl/F, apparent plasma drug clearance; V_d_/F, apparent volume of distribution; b.w., body weight. Arithmetic means ± standard deviations (SD) are shown with coefficients of variation (CV) (%) in brackets.

**Table 3:** Plasma pharmacokinetic parameters for atorvastatin, 2-OH AT, and 4-OH AT after he oral administration of a single dose of atorvastatin (20 mg/kg b.w.) to the IV_AT_ group and sorafenib+atorvastatin (100 mg/kg b.w. + 20 mg/kg b.w.) to the I_SOR+AT_ group.

Pharmacokinetic Parameters ^1^	IV_AT_ (*n* = 8)	I_SOR+AT_ (*n* = 8)	*p*-Value I_SOR+AT_ vs. IV_AT_
**Atorvastatin**			
C_max_ (ng/mL)	72.76 ± 27.21 (37.4)	81.77 ± 38.24 (46.8)	0.5957 ^1^
AUC_0-t_ (ng × h/mL)	186.70 ± 74.03 (39.7)	362.21 ± 90.82 (25.1)	0.0038 ^2^
AUC_0-∞_ (ng × h/mL)	194.01 ± 72.41 (37.3)	374.12 ± 87.23 (23.3)	0.0038 ^2^
t_max_ (h)	1.26 ± 0.69 (54.5)	2.75 ± 1.75 (63.7)	0.0419 ^1^
k_a_ (h^−1^)	1.95 ± 3.03 (155.4)	0.79 ± 0.62 (78.7)	0.1893 ^2^
k_el_ (h^−1^)	0.24 ± 0.08 (33.7)	0.34 ± 0.11 (31.6)	0.0704 ^1^
t_1/2_ (h)	3.22 ± 1.33 (41.4)	2.27 ± 0.80 (35.1)	0.1071 ^1^
Cl/F (L/h × kg)	54.31 ± 13.87 (25.5)	27.71 ± 7.46 (29.9)	0.0003 ^1^
V_d_/F (L)	257.67 ± 132.63 (51.5)	93.91 ± 50.93 (54.2)	0.0028 ^2^
**2-OH AT**			
C_max_ (ng/mL)	175.20 ± 116.62 (66.6)	185.82 ± 90.16 (48.5)	0.4948 ^2^
AUC_0-t_ (ng × h/mL)	617.32 ± 277.54 (45.0)	958.91 ± 342.91 (35.8)	0.0239 ^2^
AUC_0--∞_ (ng × h/mL)	695.68 ± 297.16 (42.7)	993.97 ± 343.87 (34.6)	0.0846 ^1^
**2-OH AT/atorvastatin**			
C_max_ (ng/mL)	2.35 ± 1.12 (47.6)	2.43 ± 1.06 (43.6)	1.0000 ^2^
AUC_0-t_ (ng × h/mL)	3.30 ± 0.70 (21.3)	2.66 ± 0.74 (27.8)	0.0520 ^2^
AUC_0-∞_ (ng × h/mL)	3.61 ± 1.12 (31.0)	2.67 ± 0.76 (28.3)	0.0312 ^2^
**4-OH AT**			
C_max_ (ng/mL)	3.18 ± 1.78 (55.9)	7.22 ± 2.42 (33.5)	0.0019 ^1^
AUC_0-t_ (ng × h/mL)	14.63 ± 5.57 (38.1)	45.26 ± 16.72 (34.9)	0.0002 ^1^
AUC_0-∞_ (ng × h/mL)	19.22 ± 7.06 (36.7)	61.59 ± 21.50 (34.9)	0.0001 ^1^
**4-OH AT/atorvastatin**			
C_max_ (ng/mL)	0.04 ± 0.02 (40.7)	0.10 ± 0.05 (50.3)	0.0084 ^1^
AUC_0-t_ (ng × h/mL)	0.08 ± 0.02 (28.3)	0.13 ± 0.06 (44.0)	0.0239 ^2^
AUC_0-∞_ (ng × h/mL)	0.10 ± 0.04 (38.1)	0.17 ± 0.08 (47.2)	0.0239 ^2^

C_max_, maximum observed plasma concentration; AUC_0-t_, area under the plasma concentration–time curve from zero to the time of last measurable concentration; AUC_0-∞_, area under the plasma concentration–time curve from zero to infinity; t_max_, time to the first occurrence of C_max_; k_a_, absorption rate constant; k_el_, elimination rate constant; t_1/2_, half-life in the elimination phase; Cl/F, apparent plasma drug clearance; V_d_/F, apparent volume of distribution; b.w., body weight. Arithmetic means ± standard deviations (SD) are shown with coefficients of variation (CV) (%) in brackets; ^1^ t-test; ^2^ Mann–Whitney U test.

**Table 4:** Plasma pharmacokinetic parameters for metformin after the oral administration of a single dose of metformin (100 mg/kg b.w.) to the V_MET_ group and sorafenib+metformin (100 mg/kg b.w. + 100 mg/kg b.w.) to the II_SOR+MET_ group.

Pharmacokinetic Parameters ^1^	V_MET_ (*n* = 8)	II_SOR+MET_ (*n* = 8)	*p*-Value II_SOR+MET_ vs. V_MET_
C_max_ (µg/mL)	0.50 ± 0.29 (58.0)	0.46 ± 0.26 (56.5)	0.9581 ^1^
AUC_0-t_ (µg × h/mL)	3.83 ± 1.23 (32.1)	3.64 ± 1.15 (31.6)	0.5635 ^1^
AUC_0-∞_ (µg × h/mL)	7.90 ± 1.93 (24.4)	10.88 ± 4.50 (41.4)	0.1035 ^1^
t_max_ (h)	1.09 ± 0.57 (52.3)	1.88 ± 0.23 (12.2)	0.0069 ^2^
k_a_ (h^−1^)	5.20 ± 5.22 (100.4)	5.06 ± 4.36 (86.2)	0.9536 ^2^
k_el_ (h^−1^)	0.03 ± 0.01 (33.3)	0.03 ± 0.04 (133.3)	0.9039 ^2^
t_1/2_ (h)	26.54 ± 14.52 (54.7)	51.51 ± 36.59 (71.0)	0.0944 ^2^
Cl/F (L/h)	6.31 ± 1.48 (23.5)	5.50 ± 3.08 (56.0)	0.1893 ^1^
V_d_/F (L)	227.66 ± 90.97 (39.9)	297.89 ± 133.23 (44.7)	0.2271 ^1^
MRT_0-t_ (h)	9.81 ± 1.27 (12.9)	8.65 ± 2.22 (25.7)	0.2202 ^2^

C_max_, maximum observed plasma concentration; AUC_0-t_, area under the plasma concentration–time curve from zero to the time of last measurable concentration; AUC_0-∞_, area under the plasma concentration–time curve from zero to infinity; t_max_, time to the first occurrence of C_max_; k_a_, absorption rate constant; k_el_, elimination rate constant; t_1/2_, half-life in the elimination phase; Cl/F, apparent plasma drug clearance; V_d_/F, apparent volume of distribution; b.w., body weight. Arithmetic means ± standard deviations (SD) are shown with coefficients of variation (CV) (%) in brackets; ^1^ t-test; ^2^ Mann–Whitney test.
